# Double-negative-2 B cells are the major synovial plasma cell precursor in rheumatoid arthritis

**DOI:** 10.3389/fimmu.2023.1241474

**Published:** 2023-08-10

**Authors:** Elinor Wing, Catherine Sutherland, Katherine Miles, David Gray, Carl S. Goodyear, Thomas D. Otto, Stefan Breusch, Graeme Cowan, Mohini Gray

**Affiliations:** ^1^ Centre for Inflammation Research, The Queen’s Medical Research Institute, University of Edinburgh, Edinburgh, United Kingdom; ^2^ Institute of Immunology and Infection Research, School of Biological Sciences, The King’s Buildings, The University of Edinburgh, Edinburgh, United Kingdom; ^3^ Institute of Infection, Immunity and Inflammation, College of Medical, Veterinary and Life Sciences, University of Glasgow, Glasgow, United Kingdom; ^4^ Orthopaedic Unit, Royal Infirmary of Edinburgh, Edinburgh, United Kingdom

**Keywords:** rheumatoid arthritis, double-negative-2 (DN2) B cells, antibody secreting cells, synovium, single-cell sequencing

## Abstract

B cells are key pathogenic drivers of chronic inflammation in rheumatoid arthritis (RA). There is limited understanding of the relationship between synovial B cell subsets and pathogenic antibody secreting cells (ASCs). This knowledge is crucial for the development of more targeted B-cell depleting therapies. While CD11c+ double-negative 2 (DN2) B cells have been suggested as an ASC precursor in lupus, to date there is no proven link between the two subsets in RA. We have used both single-cell gene expression and BCR sequencing to study synovial B cells from patients with established RA, in addition to flow cytometry of circulating B cells. To better understand the differentiation patterns within the diseased tissue, a combination of RNA-based trajectory inference and clonal lineage analysis of BCR relationships were used. Both forms of analysis indicated that DN2 B cells serve as a major precursors to synovial ASCs. This study advances our understanding of B cells in RA and reveals the origin of pathogenic ASCs in the RA synovium. Given the significant role of DN2 B cells as a progenitor to pathogenic B cells in RA, it is important to conduct additional research to investigate the origins of DN2 B cells in RA and explore their potential as therapeutic targets in place of the less specific pan-B cells depletion therapies currently in use.

## Introduction

Rheumatoid arthritis (RA) is the commonest autoimmune mediated inflammatory arthritis, affecting 1% of the world’s population. As well as chronic, severe pain and early joint destruction, patients experience marked fatigue, accelerated cardiovascular disease and premature death (1, 2). Despite the heterogenous clinical presentation, the importance of pathogenic B cells has been established and further reinforced by the success of B cell depleting therapies (BCDT) (3, 4). Pathogenic B cells contribute to chronic synovial inflammation in at least three ways; via cytokine secretion, antigen presentation, and autoantibody production (5). In RA, B cell dysregulation occurs long before clinical disease onset, and BCDT has been shown to delay the onset of established disease in pre-clinical patients with arthralgia (6, 7).

Although BCDT is beneficial in treating RA, it is non-specific, depleting all CD20^+ve^ B cells. This results in the loss of not only pathogenic B cells, but also protective B cells, which are vital for tissue homeostasis and infection control. The importance of the latter was borne out during the SARS-CoV-2 pandemic where mortality and morbidity was significantly increased in patients with autoimmunity, who had received BCDT prior to infection. In addition, the protective effect from SARS-CoV-2 vaccination was reduced in these patients (8–11). This highlights the need for more selective therapies that target only pathogenic B cells. Such therapies can only be developed when the identity of autoimmune B cells, particularly those that serve as precursors to synovial CD20^-ve^ antibody secreting cells (ASCs) is known.

Single cell analysis of the rheumatoid synovium has previously identified four B cell subsets, including naïve B cells, memory, double negative B cells and terminally differentiated antibody secreting cells (ASCs) (12). However, the inter-relatedness of these subsets is poorly understood and importantly, the major precursor to rheumatoid synovial CD20^-ve^ ASCs has not been established.

Double-negative (DN) refers to a subset of antigen experienced B cells, that lack expression of both CD27 and IgD. They are further divided into DN1 and DN2 B cells of which the latter have high expression of CD19, CD11c and the transcription factor T-bet, but lack the expression of CD21, CD24, CD38 and CXCR5 (13, 14). Whilst DN1 B cells represent the majority of DN B cells in healthy subjects, DN2 B cells are increased in the elderly, during chronic infection (including hepatitis C, malaria, and HIV) (13, 15–19) and following influenza vaccination (20, 21). Most recently they have been linked to severe and fatal outcomes from COVID-19 infection, suggesting that they have prominent pro-inflammatory functions (22). DN2 B cells are substantially increased in the circulation of patients with active systemic lupus erythematosus (SLE) and may serve as precursors to ASCs (13).

We previously observed a significant increase in the frequency of circulating class switched IgG^+ve^ DN B cells in RA patients. These B cells expressed significantly fewer mutations in the B cell receptor (BCR) and were enriched in the synovium, suggesting an extrafollicular origin (23). We considered that naïve B cells entering the synovium were induced to mature into ASCs via the generation of DN2 B cells in extrafollicular areas. We addressed this hypothesis by utilizing flow cytometry of circulating and synovial B cells as well as single cell sequencing to define the interrelatedness of the identified synovial B cell subsets.

## Results

### DN2 B cells are enriched in the blood and synovium of RA patients

**Figure 1:**
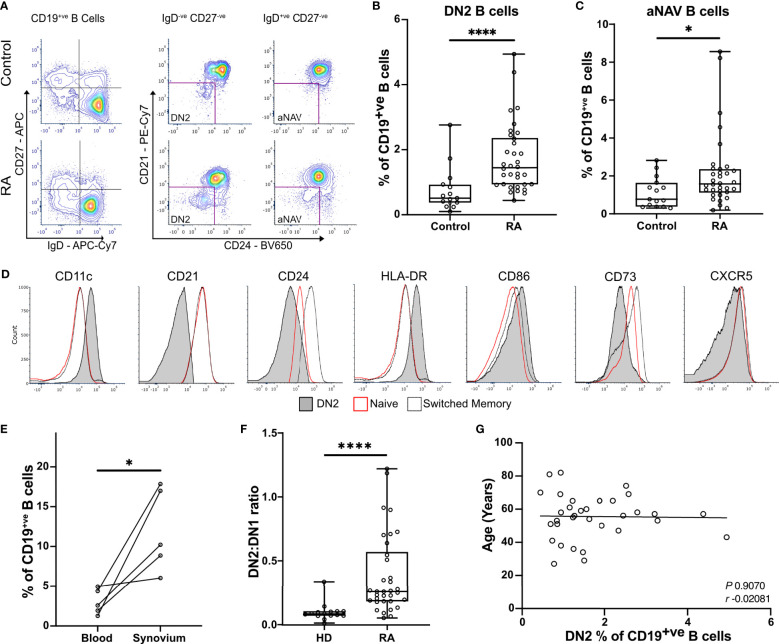
DN2 B cells are significantly enriched in the blood and synovium of RA patients. **(A)** Peripheral blood mononuclear cells (PBMCs) were stained for flow cytometry, live CD19+ve B cells were gated before selecting either the double negative (DN; CD27^-ve^ IgD^-ve^) or the naïve (CD27^-ve^ IgD^+ve^) cells. The expression of CD21, CD24, and CD38 were used to gate to double negative 2 (DN2) and activated naïve B cells (aNAV). Representative plots show the expansion of DN2 and activated naïve B cells (aNAV) in RA. **(B)** Percentage of DN2 B cells within the CD19^+ve^ B cell population. n=34 RA patients and 15 healthy controls. P<0.0001 by Mann-Whitney test. **(C)** Percentage of aNAV cells within the CD19^+ve^ B cell population. n=34 RA patients and 15 healthy controls. P=0.0329 by Mann-Whitney test. **(D)** Representative histogram plots show the expression of relevant surface markers on DN2 (filled grey), resting naïve (red line), and switched memory B cells (dotted line). **(E)** Flow cytometry of paired peripheral blood and synovium. DN2 B cells are significantly increased in the synovium of RA patients compared to blood (n=5). P=0.0257 by Paired t test. **(F)** The ratio of DN2:DN1 B cells is significantly increased as a result of DN2 cell expansion. P<0.0001 by Mann-Whitney test. * P ≤ 0.05 and **** P ≤ 0.0001. **(G)** Scatterplot of the percentage of DN2 cells and age of RA donors. Spearman’s r coefficient. * P ≤ 0.05 and **** P ≤ 0.0001.

To fully characterize rheumatoid B cells, a cohort of 34 RA patients and 15 matched healthy controls were selected for full spectrum flow cytometry (**Supplementary Table 1**). We studied all known B cell subsets in the blood (**Supplementary Table 4**). In agreement with our previous studies, DN2 B cells were increased in the circulation of RA patients when compared to healthy controls (means with 95% confidence intervals were 1.73% ± 0.37 vs 0.76% ± 0.39 P < 0.0001) (**Figures 1A, B** and **Supplementary Figure S1**). In addition, DN2 B cell precursors, called activated naïve B cells (aNAV), that are IgD^+ve^ CD27^-ve^ CD21^-ve^ CD24^-ve^ CD38^-ve^ (13), were almost doubled in frequency in the blood of RA patients compared to the healthy controls (2.19% ± 0.67 vs 1.13% ± 0.45, P=0.0329) (**Figure 1C**). The characteristic surface markers of DN2 B cells, that delineate them from both naïve and memory B cells, including CD11c, CD21, CD24 and CD73, established their full phenotype and the expression of both HLA-DR and CD86 suggested an enhanced capacity to act as APCs (**Figure 1D**). Importantly, DN2 B cells constituted a significantly higher proportion of synovial B cells compared with that of DN2 B cells in the circulating B cell population. (3.02% ± 1.97 vs. 12.0% ± 6.46, P=0.0257) (**Figure 1E**). In health, the frequency of circulating DN1 B cells predominates over DN2 B cells. However, in RA patients the ratio of circulating DN2 to DN1 B cells (percentage of DN2 B cells divided by percentage of DN1 B cells) was increased compared to healthy controls (0.39 ± 0.11 vs 0.101 ± 0.04, P<0.0001), due to the expansion of the DN2 B cell population (**Figure 1F**). The proportion of DN2 B cells in the circulation did not correlate with age, suggesting that these cells are distinct from the more broadly described age-associated B cells (ABCs) (**Figure 1G**).

### Single-cell sequencing of synovial B cells reveals the heterogeneity of B cells in the rheumatoid joint

**Figure 2:**
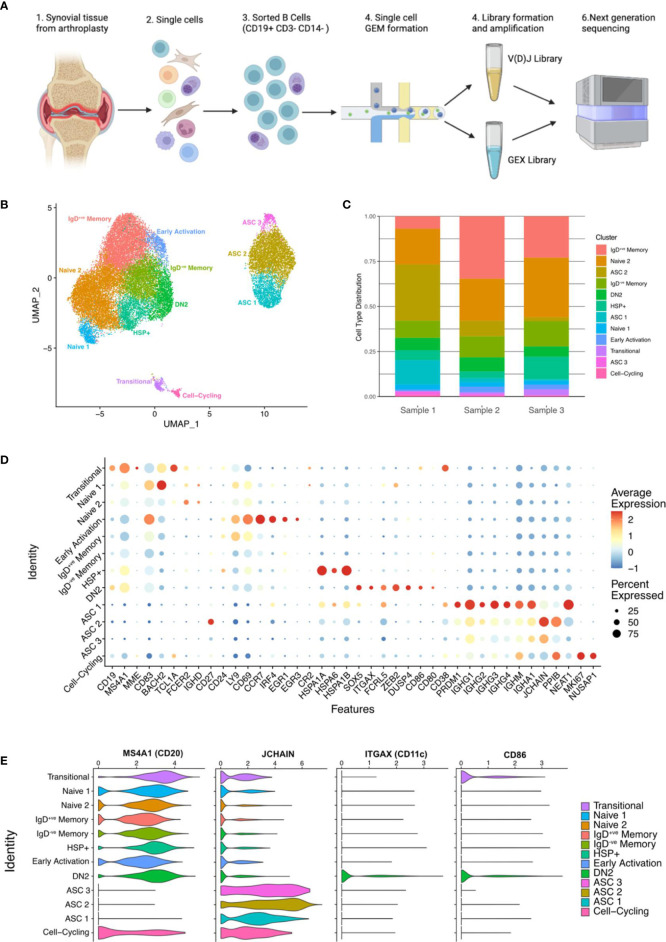
Single-cell sequencing of synovial B cells unveils the heterogeneity of B cells in the rheumatoid joint. **(A)** Overview of sample preparation: CD19+ve B cells were isolated from the synovial tissue of RA patients (n=3) before single cell RNA-sequencing with paired BCR sequencing. Created with BioRender.com. **(B)** Uniform manifold approximation and projection (UMAP) projection of all 27,053 synovial B cells from RA patients (n=3). Unsupervised clustering identified 12 clusters, the B cell subsets were annotated using the expression of known subset markers and gene signatures. DN2, double negative 2; HSP, heat shock protein; ASC, antibody secreting cell. **(C)** Stacked bar plot showing the distribution of the 12 clusters over the 3 samples. **(D)** Dotplot demonstrating the expression of key gene markers for each cluster. **(E)** Violin plots demonstrating the expression of *MS4A1* (CD20), *JCHAIN*, *ITGAX* (CD11c), and *CD86* across the 12 clusters.

Highly purified synovial B cells (CD19^+ve^ CD14^-ve^ CD3^-ve^) were isolated from the joints of 3 seropositive patients with established RA at arthroplasty (**Supplementary Figure S2A**). Single cell RNA sequencing (scRNA-seq) was carried out using the 10X Genomics Chromium platform to obtain paired transcriptomic and BCR sequence data for each cell (**Figure 2A**). In total, data for 27,053 synovial B cells were analysed, 7,825 of which had productive heavy and light chain BCR sequence information. These cells clustered into 12 distinct subsets that were annotated according to known markers and signatures (**Figure 2B**, **Supplementary Figures S2B, C**).

6.9% (1,891 of all B cells) expressed high levels of *ITGAX* (CD11c), as well as *FCRL5, CD86*, *SOX5*, and *MS4A1* (CD20) (**Figures 2C-E**), which are hallmarks of DN2 cells. Cells within this cluster lacked expression of *CD27*, *FCER2* (CD23) and *CR2* (CD21), which is consistent with the DN2 B cell population identified using full-spectrum flow cytometry.

*JCHAIN* ^+ve^ ASCs made up just over 21% of the total B cell population (5,786 cells) and encompassed three separate clusters (ASC 1, ASC 2, and ASC 3) (**Figures 2B, C**). As expected, the ASC clusters sit separately from the other B cell clusters, expressing high levels of *JCHAIN* and *PRDM1* (BLIMP1). The latter is an important regulator of ASC differentiation, that promotes the expression of *XBP1* and represses B cell transcription factors. The three ASC clusters showed distinct levels of *CD38*, *CD27*, *PRDM1* and *NEAT1* expression (**Figure 2D**). The ASC 2 cluster expresses the most *PPIB* (that forms complexes facilitating protein folding) and *JCHAIN*, both of which are involved in the unfolded protein response, that is activated during periods of high antibody production due to the immense stress on the cells. B cells from the ASC 3 cluster have the lowest average expression of ASC related genes which may suggest that they are terminally differentiated or stable ASCs that have down-regulated genes related to differentiation (**Figure 2D**).

A novel cluster of 1,708 (6.3%) B cells expressed high levels of heat shock protein (HSP) genes and are called henceforth HSP^+ve^ B cells. Although an increase in HSPs has been reported in the synovial fluid of RA patients, the subset of immune cells producing them has not been identified (24). The B cells in the HSP^+ve^ cluster expressed high levels of HSP genes including *HSPA1A*, *HSPA6*, *HSPA1B*, and *HSPB1* (**Figure 2D** and **Supplementary Figure S3A**). The high expression of HSPs is linked to the misfolded protein response and *de novo* protein folding pathways (**Supplementary Figure S3B**).

All clusters were present in each of the samples; however, the ASC clusters were primarily composed of cells from Sample 1 (68% of ASCs). The proportion of DN2 B cells was similar across the three samples, with RA1 having 6.8%, RA2 having 7.8%, and RA3 having 5.6% (**Figure 2C**).

### DN2 B cells have a transcriptional signature of antigen presentation

**Figure 3:**
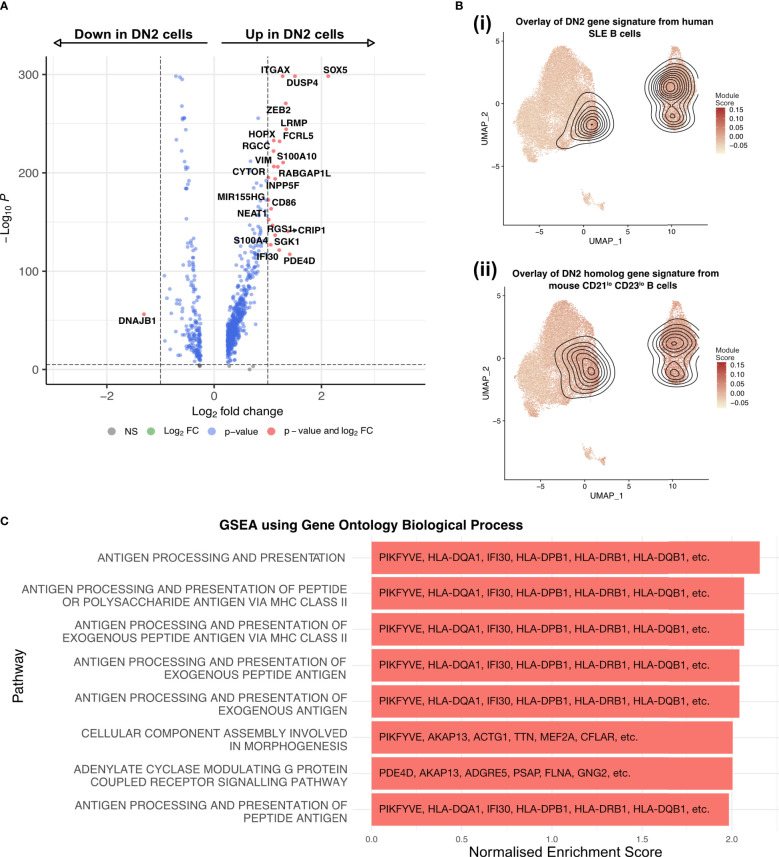
DN2 B cells are a heterogenous group of cells, primed to present antigen and become ASC. **(A)** Volcano plot showing the transcriptome analysis DEGs (red, log2FC is >|1| and P value > 10e-6; blue, log2FC is <|1| and P value > 10e-6; grey, nonsignificant genes) of DN2 B cells compared to all other non-ASC cells. **(B)** UMAP projection of scRNA-seq data from 27,053 synovial B cells from RA patients (n=3) with the module scores for (i) human SLE DN2 B cells and (ii) CD21^lo^ CD23^lo^ B cells from mice, the contour overlay represents the position of the top 10% of module scores. **(C)** The top 8 pathways enriched in the DN2 B cells as identified by gene set enrichment analysis using gene sets derived from the GO Biological Process ontology.

Differential expression analysis showed that the DN2 B cell cluster was enriched in the expression of *ITGAX* (CD11c), *SOX5*, *ZEB2*, *DUSP4*, *FCRL5*, *LRMP*, *HOPX*, *CD86* and *NEAT1*, matching previous reports of DN2 B cells in humans and CD21^lo^ B cells in mice (13, 25) (**Figure 3A**). *SOX5* is a transcription factor linked to the late stages of B cell differentiation and has previously been found to be enriched in CD21^lo^ and atypical memory B cells which share features with DN2 B cells (26). *ZEB2* has also been previously linked to autoimmune B cells as it directly binds the promoter of *ITGAX*. We compared this data to FACS sorted bulk RNA-seq data from DN2 B cells of SLE patients (13) as well as the homologous mouse CD21^lo^ CD23^lo^ B cell population (25). Projecting the location of cells with a module score in the top 10% onto the UMAP confirmed that these expression patterns overlap with the DN2 B cell cluster in this dataset, as well as the ASC1 and ASC2 clusters (**Figure 3B**). Further, GSEA analysis using the GO Biological pathways revealed strong enrichment for pathways related to antigen processing and presentation, particularly exogenous antigens via MHC Class II, due to the increased expression of CD86 and HLA genes including *HLA-DQA1*, *HLA-DQB1*, and *HLA-DRB1* (**Figure 3C**).

### Trajectory inference indicates DN2 B cells are ASC precursors in the RA synovium

**Figure 4:**
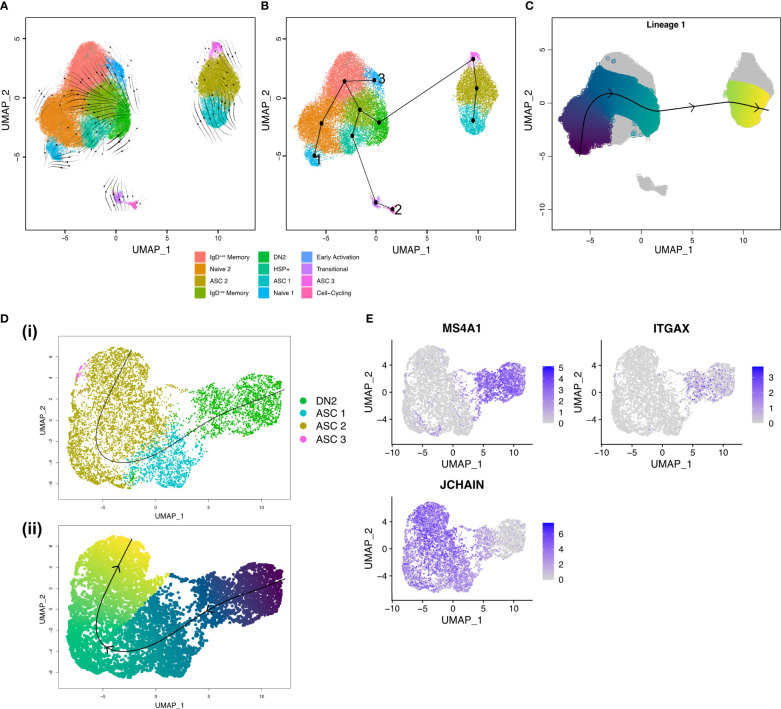
Trajectory inference shows DN2 B cells are ASC precursors in the RA synovium. **(A)** RNA velocity stream plot, projected onto the UMAP of scRNA-seq data from 27,053 synovial B cells from RA patients (n=3). **(B)** Minimum spanning tree demonstrating the full lineage structure identified by Slingshot. Lineage 1 starts in the Naïve 1 cluster, Lineage 2 in the Cell-cycling cluster, and Lineage 3 in the Early Activation cluster. **(C)** Lineage 1 identified by Slingshot with pseudotime colouring. **(D)** Slingshot trajectory identified after clustering 1,891 DN2 B cells and 5,786 ASCs from RA synovial tissue (n=3). (i) The trajectory starts in the DN2 cluster before transitioning into ASCs. (ii) The lineage identified by Slingshot with pseudotime colouring. **(E)** Expression of *MS4A1* (CD20), *ITGAX* (CD11c), and *JCHAIN* in DN2 B cells and ASCs.

As the source of autoreactive antibodies that form immune complexes, ASCs are a major contributor to inflammation and tissue damage within the joint. To delineate the developmental processes within the joint and pinpoint the main precursors of ASCs we have used a combination of RNA velocity and trajectory inference. RNA velocity analysis with scVelo (27) suggested that mature B cell clusters evolve into DN2 B cells with a subsequent onward flow towards ASC clusters (**Figure 4A**). Trajectory inference with Slingshot (28) suggested three separate lineages (**Figure 4B**). Lineage 1 starting in the Naïve 1 cluster, transitioning through the IgD^+ve^ memory B cell clusters into the IgD^-ve^ memory clusters before merging into DN2 B cells, and finally differentiating into ASCs (**Figure 4C**). Lineage 2 begins in the Cell-Cycling cluster, to IgD^-ve^ Memory, to DN2 B cells, to ASCs. Finally, Lineage 3 starts in the Early Activation cluster before joining Lineage 1 in the IgD^+ve^ Memory cluster to DN2 B cells and then to ASCs.

To gain a better understanding of the transition from DN2 B cells to ASCs, we focused our analysis on the DN2 and ASC clusters (**Figure 4D**). Upon examining the expression of MS4A1 and JCHAIN, we observed a distinct gradient indicating a decrease in CD20 expression and a simultaneous increase in JCHAIN expression as B cells potentially underwent differentiation into ASCs. The presence of such a gradient implies the possibility of a differentiation process occurring within the transition from DN2 B cells to ASCs (**Figure 4E**).

### BCR sequences from synovial B cells show hallmarks of autoimmunity

**Figure 5:**
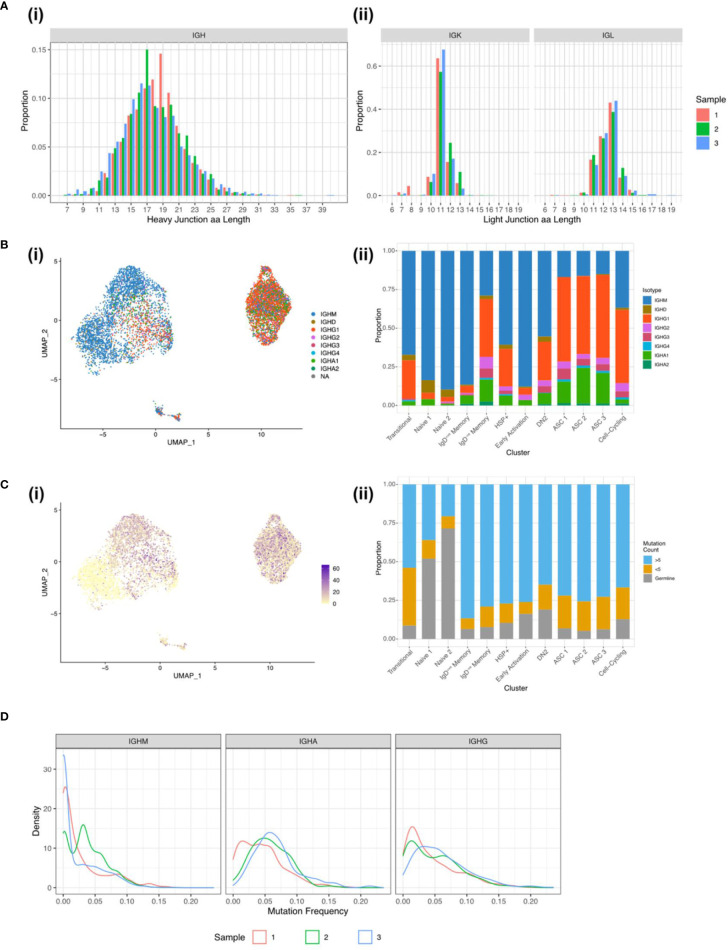
BCR sequences from synovial B cells show hallmarks of autoimmunity. **(A)** CDR3 length distribution for the (i) heavy chain and the (ii) light chains for Sample 1 (red column), Sample 2 (green column), and Sample 3 (blue column). **(B)** (i) UMAP plot of the 7,825 B cells with a productive heavy and light chain coloured by the BCR isotype. (ii) Stacked bar plot showing the distribution of the isotypes across the 12 clusters. **(C)** (i) UMAP plot of the 7,825 B cells with a productive heavy and light chain coloured by the BCR mutation count. (ii) Stacked bar plot showing the distribution of mutation counts across the 12 clusters. **(D)** Distribution of mutation frequencies for IGHM, IGHA, and IGHG for the three RA samples.

To further understand the diversity of BCRs present in the inflamed rheumatoid joint, the paired BCR repertoire sequences were evaluated, specifically looking at characteristics linked to autoimmunity. The mean IGH CDR3 lengths were similar across the three samples with Sample 1 having a mean CDR3 length of 18.3 amino acids, Sample 2 18.0, and Sample 3 17.5 amino acids (**Figure 5A**). Over a quarter (26.2%) of all IGK CDR3s are over 11 amino acids long, which has previously been linked to autoimmunity and poly-reactivity (29). As expected, the naïve clusters primarily expressed unmutated IGHM and IGHD with IGHA and IGHG being expressed in the memory and ASC clusters where class switching has occurred after activation. (**Figure 5B**). As B cells become activated both the mutation rate and proportion of class-switched cells increases, with this being evident at the border between the Naïve 2 and the memory clusters (**Figures 5B, C**). Despite the expected increase in mutation rate, unmutated sequences were present in all the clusters. In particular, 19% of the DN2 B cells expressed BCR sequences which are identical to germline sequences (**Figure 5C**). Low mutation rates persisted in some of the terminally differentiated ASCs, suggesting development out with germinal centres.

For the class-switched sequences a unimodal distribution of mutations predominates, peaking around 5% mutation frequency, but extending up to 25%. The majority of IGHM sequences remain unmutated or poorly mutated with an average mutation frequency of 3% compared to 5% in IGHG and IGHA. The sharp peak in IGHM Sample 2 comes from a large clonal expansion of an IGHM BCR sequence (**Figure 5D**).

### BCR sequences from DN2 B cells share identical CDR3 sequences with ASCs

**Figure 6:**
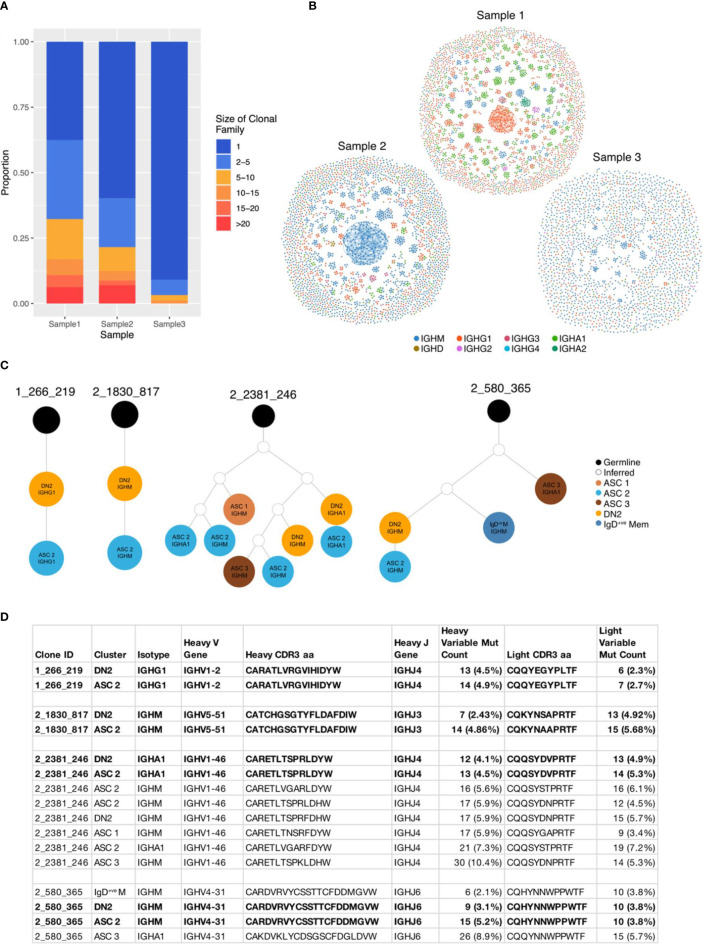
BCR sequences from DN2 B cells share identical CDR3 sequences with ASCs. **(A)** Stacked barplot showing the clonal family sizes across the three samples. **(B)** Network graphs of a representative repertoire from each RA patient. Each node represents a cell, coloured according to the isotype, with up to three edges representing the most closely related heavy CDR3 amino acid sequences according to Levenshtein distance. **(C)** Four clonal lineage trees where a DN2 cell shares an identical heavy CDR3 amino acid sequence with an ASC. Orange circles indicate a cell from the DN2 cluster, dark blue IgD^-ve^ memory, dark brown ASC 1, bright blue ASC 2, pale brown ASC 3, white inferred sequence and black is germline. **(D)** Characteristics of the 4 clonal families where a DN2 cell shares an identical junction sequence with and ASC.

We then analysed the BCR sequences in the synovium to investigate clonal relationships. The synovial B cells showed a high degree of clonal expansion, with 62.4% and 40.2% of clones being expanded (clone size ≥ 2) in Samples 1 and 2, respectively. Sample 3 was less clonal, with only 9.1% of clones consisting of at least two cells (**Figures 6A, B**). To further compare the degree of clonal expansion between the three patients we used the Gini coefficient, a measure of inequality. A Gini coefficient of 0 indicates maximal diversity or an equal abundance of each sequence, whereas 1 indicates extreme inequality. In concordance with the proportion of expanded clones, Sample 1 had the highest Gini coefficient (0.33) followed by Sample 2 (0.21), with Sample 3 having a much lower value (0.03).

There was no relationship between the size of the clonal group and the rate of mutation. The largest clonal group in Sample 1 contained 87 class switched IgG1 cells and the most mutated sequence had just 4 mutations in the heavy variable sequence, whereas a smaller group with 14 cells had up to 52 mutations (**Supplementary Figure S4A**).

Having identified (through trajectory inference and RNA velocity) that DN2 B cells are ASC precursors, we explored the relationship between the BCR sequences in these clusters. 4 clonal groups had an identical heavy and light chain CDR3 amino acid sequence between a DN2 cell and an ASC (**Figures 6C, D**). These direct links, ending at an ASC, originated from 4 DN2 cells, whereas only 1 originated in the IgD^-ve^ Memory cluster, 1 from the Naïve 2 cluster and 4 from the Cell-Cycling cluster, demonstrating that DN2 B cells are the primary precursor to ASCs (**Supplementary Figure S4B**).

## Discussion

This is the most detailed exploration to date of both circulating and synovial B cells subsets in patients with RA. Here we have shown that DN2 B cells serve as a major precursors to synovial ASCs. In addition, DN2 B cells express potent APC capacity and are therefore likely to represent an important pathogenic B cell subset that should be targeted for future therapies.

DN2 B cells play an important role in the pathogenesis of a related autoimmune rheumatic disease, SLE, where they are enriched in the blood, being epigenetically poised to respond to TLR7 ligands and suggested to serve as the pathogenic precursors to ASCs (13). We have previously observed that DN2 B cells are enriched in both the blood and synovium of RA patients (28), and confirmed this finding in a second, independent cohort of 34 patients. We found both circulating DN2 B cells and their precursors aNAVs are almost twice as frequent in RA patients. This is supported by previous reports that observe an elevation of DN cells in the blood (30, 31). This increase can be explained by a change in the dominant subtype in RA. We have found that the ratio of DN1 to DN2 B cells is reversed in RA so that the frequency of DN2 B cells as a percentage of all B cells is higher, whilst the DN1 frequency itself does not change. Hence to identify a significant elevation in pathogenic DN2 cells, all the available markers to differentiate DN1 and DN2 B cells must be included and DN B cells should not be treated as a single population.

DN2 B cells were present in the blood of healthy controls, albeit at very low numbers. A recent single cell study assessed the immune compartments of 16 tissues (not including the synovium) from 12 adult donors. They identified a B cell subset referred to as Age Associated B cells (ABCs), that has a similar transcriptional profile to the DN2 cluster from this study, including high expression of *ITGAX* and low expression of *CR2* (CD21) and *CD27* (**Supplementary Figure S5A**). They found that DN2 B cells were primarily found in the spleen, liver, and bone marrow (32). DN2 B cells may have an important role in health, but dramatically increase in autoimmune conditions and viral infections, as we have shown in RA and others have demonstrated in patients with SLE, HIV, and COVID-19 (13, 17, 22).

By assessing 27,000 synovial B cells we were able to identify 12 B cell clusters. One of these smaller clusters was entirely novel and expressed heat shock proteins (HSPs) from the HSP70 family. In RA, serum levels of HSP-70 are about twice the level of healthy controls and significantly elevated in the synovium (32). Whilst their main role is to reverse or inhibit denaturation or unfolding of cellular proteins in response to stress, they are associated with an increased frequency of Th17 T cells and the Th17/Treg ratio (33). The role that HSP^+ve^ B cells play in the pathogenesis of RA is unknown but deserves closer scrutiny in follow up studies.

Two other studies utilizing single cell sequencing of rheumatoid synovial B cells have been reported. In the first just over 1000 B cells were analyzed (from 21 subjects with established RA), and four synovial B cell subsets were identified, naïve, memory, plasmablasts and DN2 B cells (that were referred to as autoimmune B cells or ABCs in the study) (12). Of note the ABCs were derived largely from tissue samples that were leukocyte rich. We also selected samples that were leukocyte rich, so the presence of DN2 B cells may be a particular feature of this synovial pathotype and cannot necessarily be extrapolated to other histological phenotypes, such as the leukocyte poor and macrophage rich subtypes (34–36). Of note, leukocyte rich synovium is associated with the highest inflammation scores clinically and so patients with this phenotype are most likely to benefit from targeted B cell depletion (35).

A further study of newly diagnosed, untreated RA patients assessed just under 2000 B cells from 3 RA patients, 2 of whom were seronegative for antibodies directed at citrullinated autoantigens (i.e., ACPA^-ve^). Here there was no enrichment of *ITGAX^+ve^* (encoding CD11c) or CD27^-ve^ IgD^-ve^ B cells (37). The difference between these observations and our study may relate to the early stage of RA and the limited number of B cells assessed, with a predominance of ACPA^-ve^ RA donors in that study. However, our previous observation of circulating B cells in newly diagnosed, untreated RA patients clearly showed a significant increase in DN2 B cells, which suggests that even at this early stage, DN2 B cells are a prominent B cell subset and may also be expected to be enriched in the synovium (23).

A real strength of this study lies in the opportunity to understand the inter-relatedness of RA synovial B cells. RNA velocity and trajectory inference identified how the clusters of B cells exist in a dynamic continuum. We observed 3 separate lineages, beginning with naïve B cells, cell cycling B cells or the early activation cluster. Our data suggests that B cells develop into memory cells before maturing into DN2 B cells and eventually pathogenic ASCs. This implies that the RA synovium can support the generation of ASCs from three starting points; but all lineages can lead to DN2 B cells.

Previous reports allude to the heterogeneity of autoantibodies that form immune complexes within the joint (38). Similar immune complexes can be found in the joints of patients with osteoarthritis but exist at much higher frequencies in RA synovium (38). The paired BCR sequencing data has strengthened the findings of the RNA velocity analysis and provided an extra layer of detail by tracking B cell clonal lineages based on CDR3 sequence identity. Furthermore, having clonal families containing diverse combinations of B cell subsets including both naïve B cells and ASCs suggests that the immunological response is local to the joint and naïve cells are migrating to the joint where they then become activated. This is supported by another study where over 50% of the ASCs were derived from clonal families that also contained memory B cells (37).

Our data shows that, as well as differentially expressing *ITGAX*, synovial DN2 B cells highly express *DUSP4*, that encodes a member of the dual specificity protein phosphatase subfamily. DUSP4 is related to the TLR7/8 cascade through its involvement in the MyD88 pathway (39, 40). The most differentially expressed gene was *SOX5* gene, that is a member of the Sox family of transcription factors linked to cell fate (41, 42). It has previously been reported that CD11c^+ve^ B cells express both *DUSP4* and *SOX5* and our study supports that observation (43). This transcription factor decreases the proliferative capacity of B cells whilst permitting plasmablast generation (26). Hence expression of SOX5 may license the evolution of DN2 B cells into ASCs. However, we note that the expression of cytokine-related transcripts is relatively low within these synovial B cell clusters, and we did not observe a clear pattern of cytokine production among them (**Supplementary Figure S6**).

In this study we have shown that DN2 B cells are a major precursor to pathogenic ASCs in the RA synovium. We also found that they express transcriptional signatures consistent with active roles in antigen presentation and T cell activation. These results fill a gap in our understanding of B cell development in the synovial tissue of RA patients. To fully define the pathogenicity of this B cell subset the antigen specificity of these cells will need to be determined in future studies. Identifying the critical role of DN2 B cells in the generation of ASCs provides a novel target for developing future therapeutics.

## Materials and methods

### Patients

The use of human samples was approved by the South East Scotland Bioresource NHS Ethical Review Board (Ref. 15/ES/0094) and the use of healthy donor blood was approved by AMREC (Ref. 21-EMREC-041). Informed consent was obtained from all study participants prior to sample collection. The patient characteristics are described in **Supplementary Table 1**.

### Cell isolation

Peripheral blood mononuclear cells (PBMCs) were isolated from total blood using Histopaque-1077 separation according to the manufacturer’s protocol (Sigma-Aldrich). The PBMCs were collected, washed in PBS, and counted. PBMCs were resuspended in resuspension media (RPMI-1640 (Gibco) supplemented with 2 mM l-glutamine, 100 U mL^-1^ penicillin, and 100 μg mL^-1^ streptomycin, with 40% fetal calf serum (FCS)) then an equal volume of freezing media (resuspension media supplemented with 30% dimethyl sulfoxide (DMSO, Fisher Reagents)) was added to give final density of 10x10^6^ cells mL^−1^ in 15% DMSO. The sample was then frozen to -80°C in a Mr Frosty Freezing Container (ThermoFisher Scientific) then stored in liquid nitrogen until further use.

Synovial tissue was collected from RA patients undergoing arthroplasty. The fresh tissue was dissected followed by digestion for 2 hours at 37°C in 1 mg mL^−1^ Collagenase 1 (Sigma-Aldrich). Debris was removed and cells isolated by sequentially passing through 100, 70 and 40μm cell strainers (Corning). The isolated cells were then frozen and stored in the same way as the PBMCs.

### Full spectrum flow cytometry

PBMC and synovial tissue samples were thawed and resuspended according to the 10X protocol (10X Genomics). Briefly, cryovials were removed from liquid nitrogen and thawed rapidly in a water bath at 37°C. The cells were sequentially diluted 5 times with warm media with 1:1 incremental volume addition added in a dropwise manner, gently swirling between each volume. The cell suspension was centrifuged at 230xg for 6 minutes then washed in PBS. A 40μm cell strainer was used to remove debris before the cells were washed and stained in FACS buffer (PBS supplemented with 1% FCS) at room temperature for 20 minutes with fluorescently conjugated antibodies as described in **Supplementary Table 2**. Cells were washed and re-suspended in FACS buffer and analysed using a Cytek Aurora (Cytek Biosciences) and FCS Express (v7.14, *De Novo* Software).

### 10X run and sequencing

Synovial tissue samples were thawed and resuspended according to the 10X protocol as described above. Cells were washed and then stained in FACS buffer at 4°C for 20 minutes with fluorescently conjugated antibodies as described in **Supplementary Table 3**. Single CD19^+ve^ DAPI^-ve^ CD3^-ve^ CD14^-ve^ B cells were then sorted with the FACS Aria II and FACS Diva 8.0.1 (BD Biosciences). The full gating strategy is shown in **Supplementary Figure S2A**. Immediately after sorting the CD19^+ve^ B cells were processed through the Chromium Single Cell Platform using the Chromium Next GEM Single Cell 5’ Kit v2 (10X Genomics, PN- 1000265), the Chromium Single Cell Human BCR Amplification Kit (10X Genomics, PN-1000253), and the Chromium Next GEM Chip K Single Cell Kit (10X Genomics, PN-1000287) as per manufacturer’s protocol. GEX and V(D)J libraries were then sequenced using the NextSeq2000 at the Wellcome Trust Clinical Research Facility’s Genetics Core in Edinburgh.

### Data pre-processing

The FASTQ files were processed using Cell Ranger (10X Genomics, v7.0.0) and aligned to the GRCh38 reference genome using the cellranger multi command. The raw feature matrices were loaded into R (v4.1.0) using the Seurat (v4.1.0) package (44). Each sample was pre-processed separately before integration using Harmony (v0.1) (45). Cells that expressed fewer than 200 distinct genes, more than 2500 distinct genes, or had more than 5% mitochondrial reads were removed in order to exclude potential multiplets and dead cells. Contaminating non-B cells were then excluded by removing cells expressing *CD3E*, *CD14*, *GNLY*, *FCER1A*, *GCGR3A* (*CD16*), *LYZ*, or *PPBP*. Following quality control, the three samples had 9,044, 11,840, and 6,169 cells. Gene counts were then log-normalized with default parameters and the top 2,000 variable features for each sample were identified using Seurat’s FindVariableFeatures function. To prevent expression of genes associated with individual BCR clonotypes from influencing dimensionality reduction and clustering, immunoglobulin related genes, including all heavy and light VDJ genes and constant region genes, were removed from the variable features list (1761 variable features remained).

### Clustering, dimensionality reduction, and differential expression

Unsupervised clustering based on the first 20 principal components of the most variably expressed genes was performed using Seurat::FindNeighbours and Seurat::FindClusters with the resolution 0.5. Clusters were visualized using the manifold approximation and projection (UMAP) method and annotated by analyzing the expression of canonical cell markers, SingleR (46), and the use of pre-made gene sets from bulk RNA-seq of sorted B cell populations downloaded from MSigDB (v7.5.1) (47). Differential gene expression between clusters was examined using the MAST test using Seurat::FindMarkers, the results are reported as volcano plots created with EnhancedVolcano (v1.1).

### Module scores and gene set enrichment analysis

Module scores were created using the Seurat::AddModuleScore function. The Human SLE DN2 gene signature was generated using the normalized gene expression data from GSE92387 (15), the data was downloaded and analyzed using the SARtools R package (48). The top DEGs with >1.5 log2FC were then used as the input list for Seurat::AddModuleScore creating a module score for each cell. The mouse CD21^lo^ CD23^lo^ gene signature was generated using the differentially expressed genes in CD21^lo^ CD23^lo^ cells relative to follicular B cells (full gene list provided in personal correspondence) (19). The module score was created as before but with the top DEGs with >2.5 log2FC. The module scores were then used as features in Seurat::FeaturePlot, and contour plots layered over to show the cells within the top 10% for each module score.

Gene set enrichment analysis (GSEA) was carried out using fgsea (v1.21) (49). Gene Ontology (GO) gene sets were downloaded from MSigDB (v7.5.1). The differentially expressed genes identified by Seurat::FindMarkers were ranked by log2 fold change and used as the input. The GSEA results were reported with the normalized enrichment score (NES).

### Trajectory inference and RNA velocity

Trajectory inference was performed using Slingshot (v2.2) (28). Slingshot uses the clusters identified by Seurat in an unsupervised manner to construct a minimum spanning tree which is then converted into smooth lineages aligning cells to a pseudotime trajectory.

RNA Velocity analysis was performed using Velocyto (v0.17) (50) and scVelo (v 0.2.4) (27). Velocyto was used to identify spliced and unspliced transcripts within the Cellranger output. The resulting loom output file was then merged with the cluster annotation and dimensionality reduction metadata, previously generated in the Seurat analysis, using scVelo. RNA velocities were estimated using the dynamical model from scVelo and projected onto the UMAP.

### BCR analysis

BCR repertoire data were processed using the Immcantation framework. Initial VDJ assignment was carried out using IgBLAST (v1.18.0) (51) with the aid of the AssignGenes.py wrapper and output was formatted using MakeDb.py, both scripts from Change-O (52). Clustering into clonal groups was also carried out using Change-O. Clones were defined as having identical VJ gene usage and junction length with a normalised Hamming distance threshold of 0.2, as determined by the distToNearest function in the SHazaM R package, used to further partition sequences (**Supplementary Figure S7**). Germline sequences for each clonal group were reconstructed using CreateGermlines.py from Change-O. The R package Alakazam was used for clonal lineage reconstruction, lineage topology and repertoire diversity analysis. Mutation counts and frequencies were calculated using a custom Python script, mutation frequency was defined as number of mutations divided by length of sequence. The BCR data, including the mutational loads and clonal assignments, were then integrated with the processed gene expression data in Seurat. Input for the network graphs was generated with custom Python scripts and visualized using Gephi (v0.9.7).

### Statistics

Data were assessed for normality using the Shapiro-Wilk normality test before the appropriate parametric or non-parametric test was chosen. All paired and unpaired Student’s T-tests, Mann-Whitney tests, and Spearman correlations for the full-spectrum flow cytometry data were performed using Prism 9.4 (GraphPad Software Inc.). Use of the ± following mean values indicates the 95% confidence interval.

## Data availability statement

The datasets presented in this study can be found in online repositories. The names of the repository/repositories and accession number(s) can be found below: E-MTAB-12833 (Array Express).

## Ethics statement

The use of human samples was approved by the South East Scotland Bioresource NHS Ethical Review Board (Ref. 15/ES/0094) and the use of healthy donor blood was approved by AMREC (Ref. 21-EMREC-041). The studies were conducted in accordance with the local legislation and institutional requirements. The participants provided their written informed consent to participate in this study.

## Author contributions

MG, EW, KM and GC designed and/or carried out experiments. EW, CS and GC undertook bioinformatics analysis. MG conceived of project, obtained grant funding, and with EW, wrote the manuscript.
