# Supplementary material for: Double-negative-2 B cells are the major synovial plasma cell precursor in rheumatoid arthritis
Source: Front Immunol. 2023 Aug 10;14:1241474. doi: 10.3389/fimmu.2023.1241474 (PMC10450142; doi:10.3389/fimmu.2023.1241474)
Supplement: Supplementary file 1 [file Table_1.pdf]

Supplementary Tables

Supplementary Table 1 – Characteristics of patients used for full spectrum flow cytometry and the scRNA-seq.

	RA patients for flow cytometry (n=34)	Paired synovium for flow cytometry (n=5)	Arthroplasty patients for single cell RNA-seq (n=3)
Age (year, IQR)	57.5 (50.3-65)	58.9 (49.8-68.6)	66.8 (66.6-68.1)
Female (%)	25 (73.5)	4 (80)	3 (100)
CCP (IU/ml, IQR)	164.8 (36-238) *[1]	219 (105.5-323) *[4]	473 (336.6-536.5)
RF (IU/ml, IQR)	149 (27.5-223) *[2]	314.72 (200-500)	326.5 (263.3-389.75) *[5]
Baseline DAS28 (IQR)	5.26 (3.36-6.93) *[3]	N/A	N/A
DMARD treatment			
-Methotrexate	9	2	1
-Hydroxychloroquine	6	1	0
-Sulfasalazine	4	2	0
-Leflunomide	1	0	0
Biologic Therapies			
-Adalimumab	0	0	1
-Etanercept	1	1	0
Number of concurrent DMARDS			
-One	8	1	1
-Two	2	2	0
-Three	1	0	0

*[1] n = 25, *[2] n = 26, *[3] n = 28, *[4] n = 3, *[5] n = 2.
