# Supplementary material for: Double-negative-2 B cells are the major synovial plasma cell precursor in rheumatoid arthritis
Source: Front Immunol. 2023 Aug 10;14:1241474. doi: 10.3389/fimmu.2023.1241474 (PMC10450142; doi:10.3389/fimmu.2023.1241474)
Supplement: Supplementary file 2 [file Table_2.pdf]

Supplementary Table 2 - Antibody reagents for FACS sorting.

Antibody (Clone)	Fluorochrome	Isotype	Source	Dilution
Anti-human CD19 (HIB19)	PE	Mouse IgG1, κ	BioLegend	1:50
Anti-human CD3 (HIT3a)	FITC	Mouse IgG2a, κ	BioLegend	1:50
Anti-human CD14 (63D3)	APC	Mouse IgG1, κ	BioLegend	1:50
DAPI			Sigma-Aldrich	1:10000
