# Supplementary material for: Double-negative-2 B cells are the major synovial plasma cell precursor in rheumatoid arthritis
Source: Front Immunol. 2023 Aug 10;14:1241474. doi: 10.3389/fimmu.2023.1241474 (PMC10450142; doi:10.3389/fimmu.2023.1241474)
Supplement: Supplementary file 3 [file Table_3.pdf]

Supplementary Table 3 - Antibodies used in full spectrum flow cytometry staining.

Antibody (Clone)	Fluorochrome	Isotype	Source	Panel	Dilution
Anti-human CD10 (HI10a)	Brilliant Violet 480	Mouse IgG1, κ	BD Biosciences	1 & 2	1:67
Anti-human CD11c (3.9)	Brilliant Violet 785	Mouse IgG1, κ	BioLegend	1 & 2	1:50
Anti-human CD138 (1D4)	PE-Cy5	Mouse IgG1	Stratech	1	1:50
Anti-human CD138 (MI15)	PE/Cy5	Mouse IgG1, κ	AAT Bioquest	2	1:50
Anti-human CD14 (63D3)	BV510	Mouse IgG2a, κ	BioLegend	1	1:50
Anti-human CD184 (CXCR4) (12G5)	PerCP-eFluor™ 710	Mouse IgG2a, κ	ThermoFisher Scientific	2	1:50
Anti-human CD185 (CXCR5) (MU5UBEE)	Super Bright 436	Mouse IgG2b, κ	ThermoFisher Scientific	1 & 2	1:50
Anti-human CD19 (HIB19)	Brilliant Violet 711	Mouse IgG1, κ	BioLegend	2	1:50
Anti-human CD19 (HIB19)	PE/Dazzle™ 594	Mouse IgG1, κ	BioLegend	1	1:50
Anti-human CD20 (2H7)	eFluor 450	Mouse IgG2b, κ	ThermoFisher Scientific	1 & 2	1:50
Anti-human CD21 (Bu32)	PE/Cyanine7	Mouse IgG1, κ	BioLegend	1 & 2	1:50
Anti-human CD23 (M-L233)	Brilliant Violet 750	Mouse IgG1, κ	BD Biosciences	1 & 2	1:67
Anti-human CD24 (ML5)	Brilliant Violet 650	Mouse IgG2a, κ	BD Biosciences	1 & 2	1:50
Anti-human CD27 (M-T271)	APC	Mouse IgG1, κ	BD Biosciences	2	1:20
Anti-human CD3 (UCHT1)	Alexa Fluor 532	Mouse IgG1, κ	ThermoFisher Scientific	1 & 2	1:50
Anti-human CD307e (FcRL5) (509f6)	PE	Mouse IgG2a, κ	BioLegend	1 & 2	1:50
Anti-human CD38 (HIT2)	Brilliant Violet 605	Mouse IgG1, κ	BioLegend	1 & 2	1:50
Anti-human CD39 (A1)	PE/Fire™ 810	Mouse IgG1, κ	BioLegend	2	1:50
Anti-human CD40 (5C3)	Alexa Fluor 700	Mouse IgG1, κ	BioLegend	2	1:50
Anti-human CD45RB (MT4)	BV711	Mouse IgG1, κ	BD	1	1:100
Anti-human CD5 (L17F12)	PE/Dazzle™ 594	Mouse IgG2a, κ	BioLegend	2	1:50
Anti-human CD73 (Ecto-5'-nucleotidase) (AD2)	FITC	Mouse IgG1, κ	BioLegend	1 & 2	1:50
Anti-human CD86 (BU63)	Brilliant Violet 421	Mouse IgG1, κ	BioLegend	1 & 2	1:50

Anti-human CD95 (Fas) (DX2)	PE/Fire™ 640	Mouse IgG1, κ	BioLegend	2	1:50
Anti-human CD95 (Fas) (DX2)	AF700	Mouse IgG1, κ	BioLegend	1	1:50
Anti-human HLA-DR (L243)	APC/Fire™ 810	Mouse IgG2a, κ	BioLegend	1 & 2	1:50
Anti-human IgA (IS11-8E10)	VioGreen	Mouse IgG1, κ	Miltenyi Biotec	2	1:100
Anti-human IgD (IA6-2)	APC/Fire™ 750	Mouse IgG2a, κ	BioLegend	2	1:50
Anti-human IgD (IA6-2)	APC-Cy7	Mouse IgG2a, κ	BioLegend	1	1:50
Anti-human IgG (M1310G05)	Alexa Fluor 647	Rat IgG2a, κ	BioLegend	1 & 2	1:50
Anti-human IgM (MHM-88)	Brilliant Violet 570	Mouse IgG1, κ	BioLegend	2	1:50
Anti-human IgM (SA-DA4)	PerCP-eF710	Mouse IgG1, κ	ThermoFisher	1	1:50
Fixable Viability Kit	Zombie NIR™		BioLegend	1 & 2	1:1000
