# Supplementary material for: Double-negative-2 B cells are the major synovial plasma cell precursor in rheumatoid arthritis
Source: Front Immunol. 2023 Aug 10;14:1241474. doi: 10.3389/fimmu.2023.1241474 (PMC10450142; doi:10.3389/fimmu.2023.1241474)
Supplement: Supplementary file 4 [file Table_4.pdf]

Supplementary Table 4 - Subset proportions for all known B cell subsets in the blood. Mann-Whitney test for non-normally distributed, unpaired data. t test for normally distributed, unpaired data.

B Cell Population		Markers	Healthy (%, IQR) n=15	RA (%, IQR) n=29	P value
Naive		IgD^{+ve} CD27^{-ve}	70.3 (14.4)	72.3 (8.2)	0.5837
Unswitched Memory		IgD^{+ve} CD27^{+ve}	12.3 (8.83)	10.3 (5.54)	0.4188
Switched Memory		IgD^{-ve} CD27^{+ve}	13.4 (6.9)	13.2 (6.9)	0.9233
Double Negative		IgD^{-ve} CD27^{-ve}	11.4 (5.25)	13.5 (5.0)	0.2721
Transitional	T1	IgD^{+ve} CD27^{-ve} CD38^{high} CD24^{high}	3.68 (2.04)	3.58 (1.9)	0.8871
	T2-MZP	IgD^{+ve} CD27^{-ve} CD38^{high} CD24^{high} CD21^{high}	0.227 (0.27)	0.226 (0.27)	0.3934
	T3	IgD^{+ve} CD27^{-ve} CD38^{+ve} CD24^{+ve} CD21^{+ve}	1.75 (1.95)	1.67 (1.35)	0.6727
Naive	Resting	CD38^{+ve} CD24^{+ve} CD21^{+ve}	48.6 (13.5)	47.1 (13.1)	0.7772
	Activated	CD38^{-ve} CD24^{-ve} CD21^{-ve}	1.13 (0.889)	2.35 (1.52)	**0.0091**
	Anergic	CD38$^{+ve/low}$ CD24^{+ve} CD21^{-ve}	6.61 (4.0)	5.79 (2.2)	0.6773
Memory	Unswitched	CD38$^{+ve/low}$ CD24^{+ve} CD21^{+ve}	10.1 (8.2)	8.09 (4.6)	0.3139
	IgM^{+ve} Only	IgD^{-ve} IgM^{+ve} CD27^{+ve} CD38$^{+ve/low}$ CD24^{+ve} CD21^{+ve}	2.86 (2.1)	1.32 (0.718)	**0.0008**
	Switched Resting	CD38$^{+ve/low}$ CD24^{+ve} CD21^{+ve}	6.92 (4.92)	5.36 (3.5)	0.2855
	Switched Active	CD38^{-ve} CD24^{-ve} CD21^{-ve}	0.607 (0.50)	1.24 (1.07)	0.0943
Double Negative	DN1	CD38^{+ve} CD24^{+ve} CD21^{+ve}	7.28 (3.9)	6.78 (3.94)	0.3479
	DN2	CD38^{-ve} CD24^{-ve} CD21^{-ve}	0.757 (0.77)	1.51 (0.59)	**0.0003**
ASC		CD27^{high} CD20^{-ve}	1.33 (0.92)	2.43 (2.0)	0.5278
B1		IgD^{high} CD27^{+ve}	0.691 (0.92)	0.933 (0.81)	0.6027
