# Supplementary material for: Double-negative-2 B cells are the major synovial plasma cell precursor in rheumatoid arthritis
Source: Front Immunol. 2023 Aug 10;14:1241474. doi: 10.3389/fimmu.2023.1241474 (PMC10450142; doi:10.3389/fimmu.2023.1241474)
Supplement: Supplementary file 5 [file Image_1.pdf]

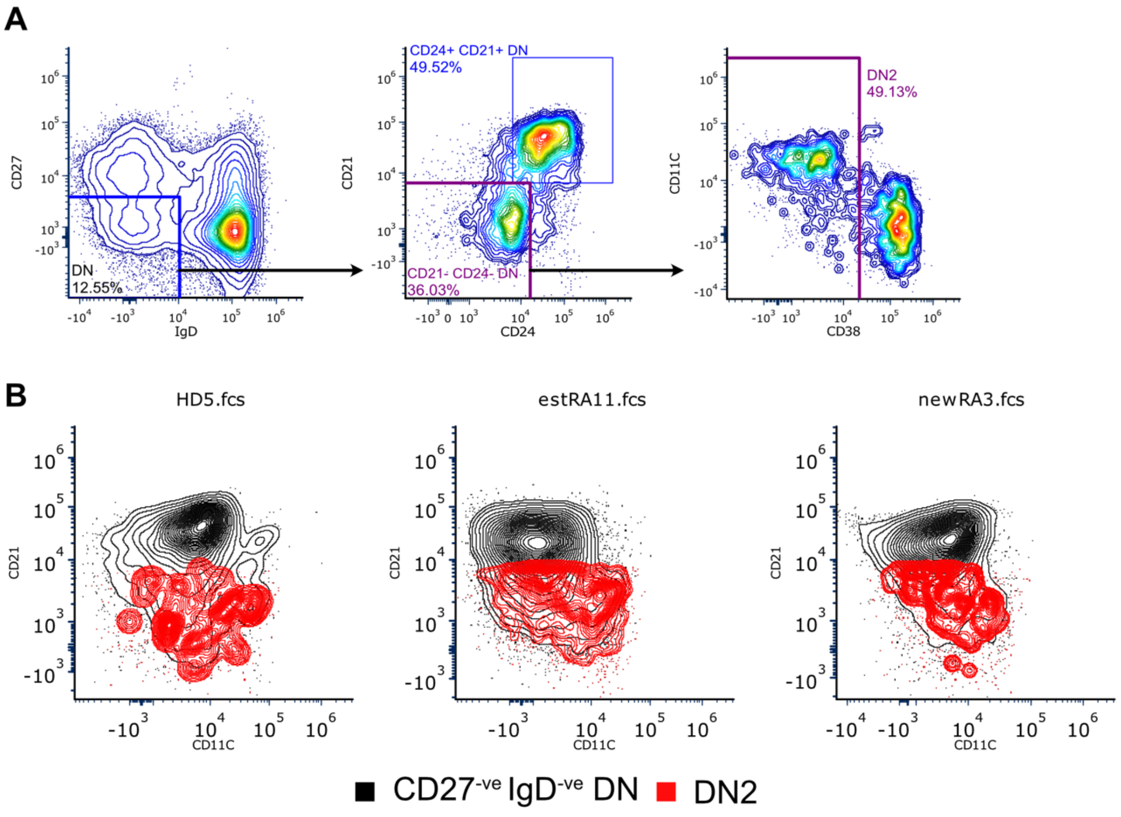

Supplementary Figure S1: DN2 gating strategy.

A) Flow cytometry gating strategy for DN2 B cells in Figure 1. From CD19^{+ve} CD3^{-ve} B cells the CD27^{-ve} IgD^{-ve} DN population was gated, from the DN population theCD21^{-ve} CD24^{-ve} were gated followed by the CD38^{-ve}.

B) Representative contour plots of CD21 and CD11c expression within the DN population with colour overlay for the gated DN2 population.
