# Supplementary material for: Double-negative-2 B cells are the major synovial plasma cell precursor in rheumatoid arthritis
Source: Front Immunol. 2023 Aug 10;14:1241474. doi: 10.3389/fimmu.2023.1241474 (PMC10450142; doi:10.3389/fimmu.2023.1241474)
Supplement: Supplementary file 6 [file Image_2.pdf]

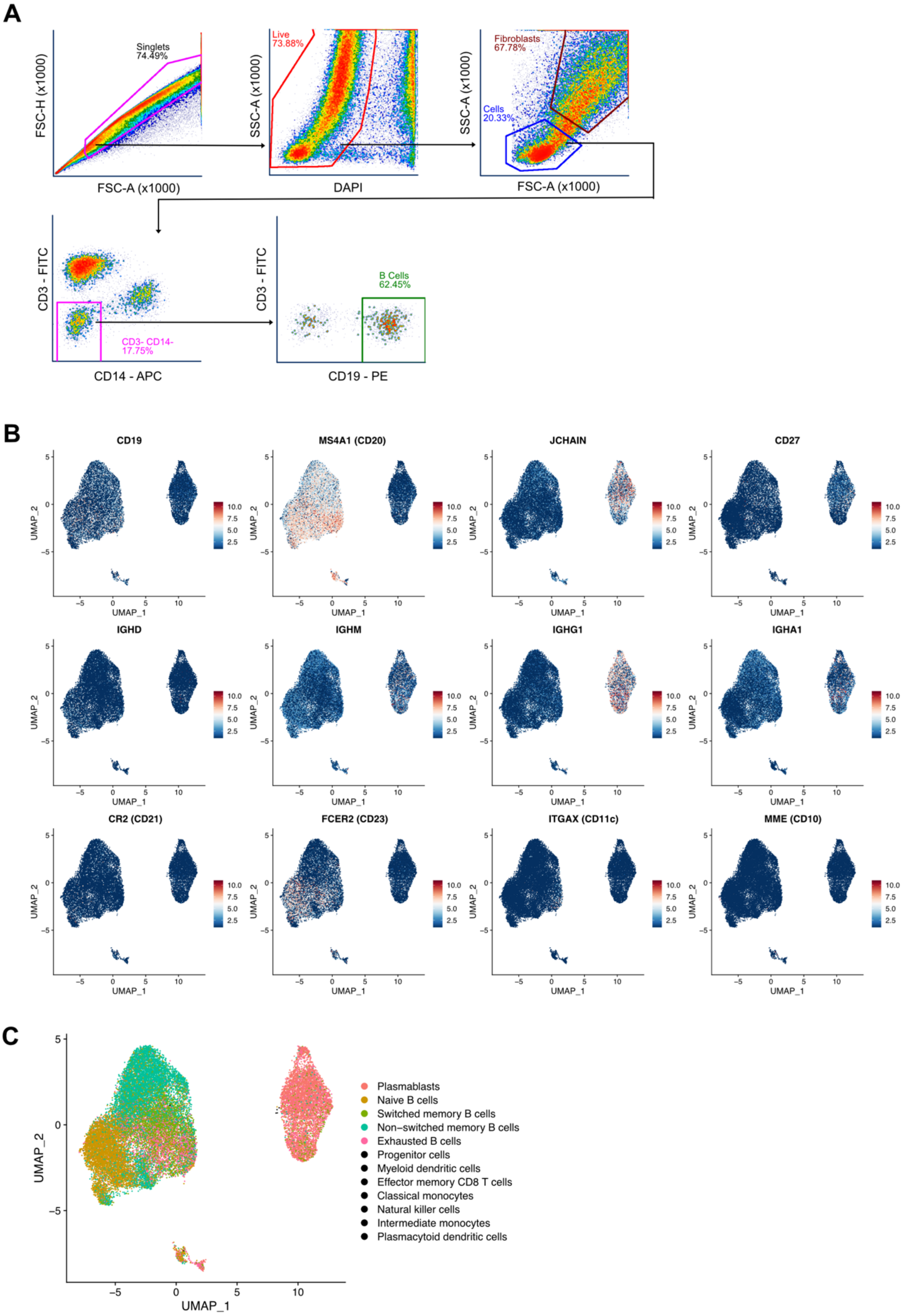

Supplementary Figure S2: Isolation and annotation of CD19^{+ve} synovial B cells.

A) Flow cytometry gating strategy for FACS sorting CD19^{+ve} B cells from human synovial tissue; representative plot from three samples.

B) Selected expression of important genes used for identifying B cell subsets.

C) Annotation from SingleR Monaco Immune Data generated from adult peripheral blood B cell signatures.
