# Supplementary material for: Double-negative-2 B cells are the major synovial plasma cell precursor in rheumatoid arthritis
Source: Front Immunol. 2023 Aug 10;14:1241474. doi: 10.3389/fimmu.2023.1241474 (PMC10450142; doi:10.3389/fimmu.2023.1241474)
Supplement: Supplementary file 7 [file Image_3.pdf]

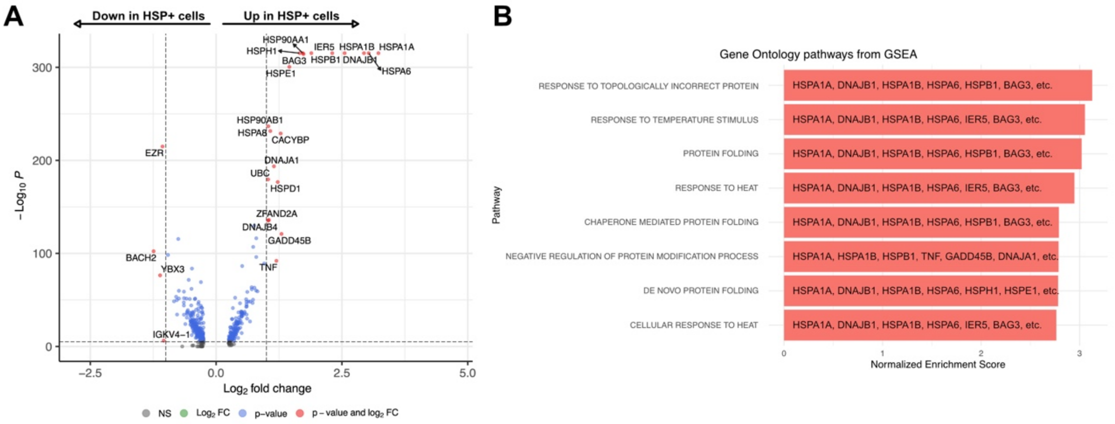

Supplementary Figure S3: Heat shock protein cluster

A) Differential expression analysis of HSP+ cells compared to all other non-ASCs.

B) Gene set enrichment analysis using GO Biological Pathways gene sets.
