# Supplementary material for: Double-negative-2 B cells are the major synovial plasma cell precursor in rheumatoid arthritis
Source: Front Immunol. 2023 Aug 10;14:1241474. doi: 10.3389/fimmu.2023.1241474 (PMC10450142; doi:10.3389/fimmu.2023.1241474)
Supplement: Supplementary file 8 [file Image_4.pdf]

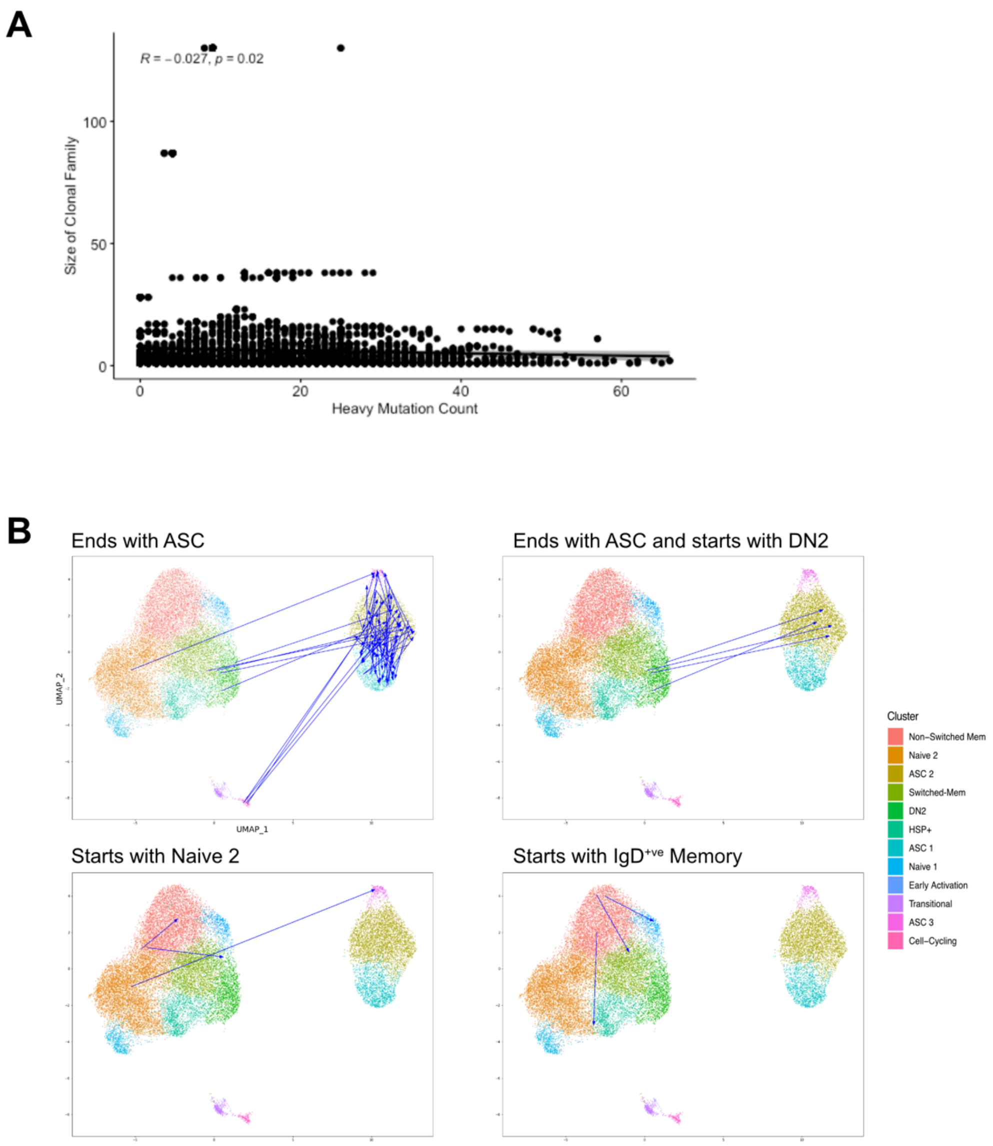

Supplementary Figure S4: BCR

A) There was no relationship between the size of the clonal family and the rate of mutation.

B) UMAP with direct relationships, where the direct link indicates an identical heavy CDR3 amino acid sequence between the cells. Here we show all direct relationships that end with an ASC, relationships that end with an ASC but start with a DN2 B cell, direct relationships starting with a Naïve 2 B cell, and direct relationships starting with an IgD^{-ve} Memory B cell. The direct relationships from Naïve 2 and IgD^{-ve} Memory cells compliment the trajectory patterns identified by both Slingshot and RNA velocity.
