# Supplementary figures and images for: Double-negative-2 B cells are the major synovial plasma cell precursor in rheumatoid arthritis
Source: Front Immunol. 2023 Aug 10;14:1241474. doi: 10.3389/fimmu.2023.1241474 (PMC10450142; doi:10.3389/fimmu.2023.1241474)

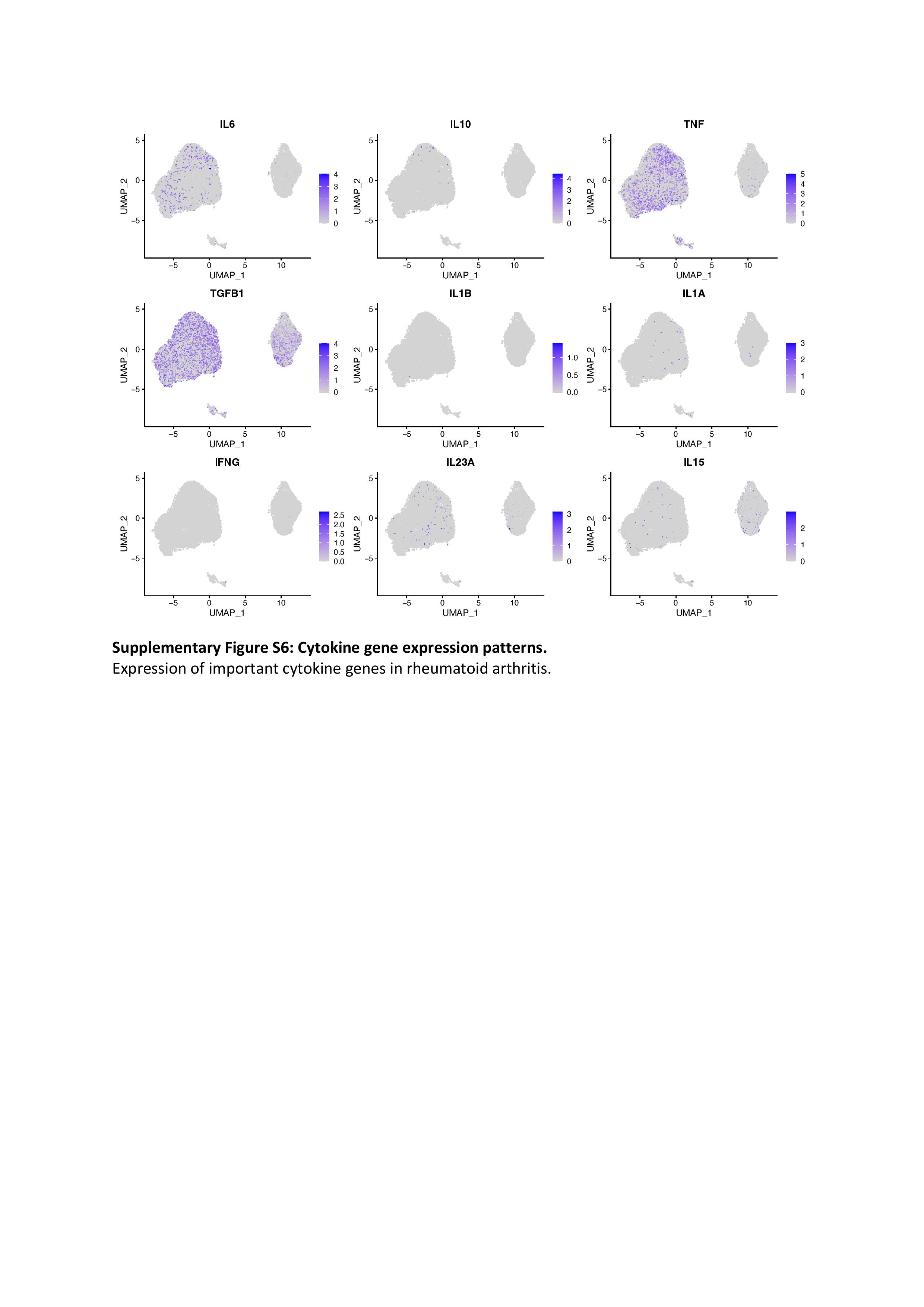

Supplement: Supplementary file 10 [file Image_6.jpg]
