# Supplementary material for: Double-negative-2 B cells are the major synovial plasma cell precursor in rheumatoid arthritis
Source: Front Immunol. 2023 Aug 10;14:1241474. doi: 10.3389/fimmu.2023.1241474 (PMC10450142; doi:10.3389/fimmu.2023.1241474)
Supplement: Supplementary file 11 [file Image_7.pdf]

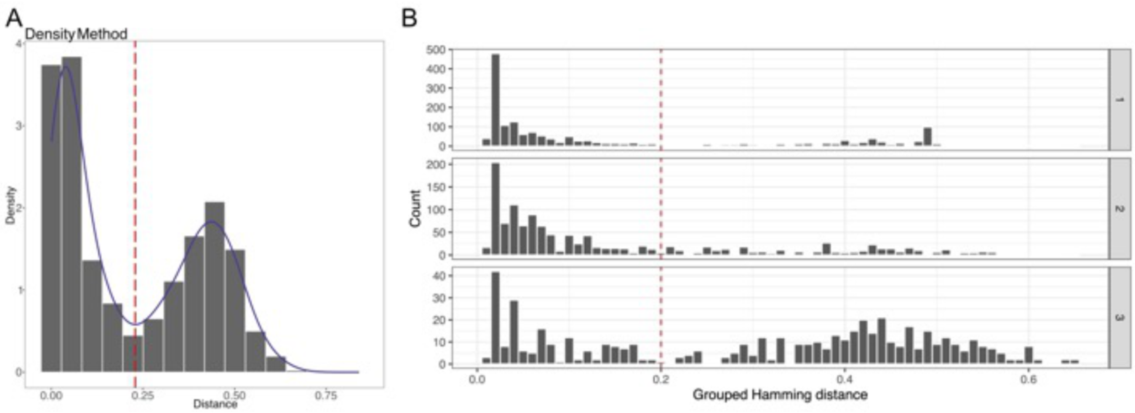

Supplementary Figure S7: Calculating hamming distance for Change-O.

A) Shazam output histogram of the nearest neighbour distances followed by automated threshold detection for the threshold separating the two modes.

B) Histogram of the nearest neighbour independently for each sample.
